# Anti-replicative recombinant 5S rRNA molecules can modulate the mtDNA heteroplasmy in a glucose-dependent manner

**DOI:** 10.1371/journal.pone.0199258

**Published:** 2018-06-18

**Authors:** Romuald Loutre, Anne-Marie Heckel, Damien Jeandard, Ivan Tarassov, Nina Entelis

**Affiliations:** UMR 7156 Génétique Moléculaire, Génomique, Microbiologie (GMGM), Strasbourg University-CNRS, Strasbourg, France; Radboudumc, NETHERLANDS

## Abstract

Mutations in mitochondrial DNA are an important source of severe and incurable human diseases. The vast majority of these mutations are heteroplasmic, meaning that mutant and wild-type genomes are present simultaneously in the same cell. Only a very high proportion of mutant mitochondrial DNA (heteroplasmy level) leads to pathological consequences. We previously demonstrated that mitochondrial targeting of small RNAs designed to anneal with mutant mtDNA can decrease the heteroplasmy level by specific inhibition of mutant mtDNA replication, thus representing a potential therapy. We have also shown that 5S ribosomal RNA, partially imported into human mitochondria, can be used as a vector to deliver anti-replicative oligoribonucleotides into human mitochondria. So far, the efficiency of cellular expression of recombinant 5S rRNA molecules bearing therapeutic insertions remained very low. In the present study, we designed new versions of anti-replicative recombinant 5S rRNA targeting a large deletion in mitochondrial DNA which causes the KSS syndrome, analyzed their specific annealing to KSS mitochondrial DNA and demonstrated their import into mitochondria of cultured human cells. To obtain an increased level of the recombinant 5S rRNA stable expression, we created *trans*mitochondrial cybrid cell line bearing a site for Flp-recombinase and used this system for the recombinase-mediated integration of genes coding for the anti-replicative recombinant 5S rRNAs into nuclear genome. We demonstrated that stable expression of anti-replicative 5S rRNA versions in human *trans*mitochondrial cybrid cells can induce a shift in heteroplasmy level of KSS mutation in mtDNA. This shift was directly dependent on the level of the recombinant 5S rRNA expression and the sequence of the anti-replicative insertion. Quantification of mtDNA copy number in transfected cells revealed the absence of a non-specific effect on wild type mtDNA replication, indicating that the decreased proportion between mutant and wild type mtDNA molecules is not a consequence of a random repopulation of depleted pool of mtDNA genomes. The heteroplasmy change could be also modulated by cell growth conditions, namely increased by cells culturing in a carbohydrate-free medium, thus forcing them to use oxidative phosphorylation and providing a selective advantage for cells with improved respiration capacities. We discuss the advantages and limitations of this approach and propose further development of the anti-replicative strategy based on the RNA import into human mitochondria.

## Introduction

Mitochondria are essential organelles of human cells because of their fundamental roles in several critical cellular processes including energy generation, Fe-S clusters production, calcium homeostasis and apoptosis. They contain their own genome, in multiple copies per cell, allowing the synthesis of 13 polypeptides which are all essential components of the mitochondrial oxidative phosphorylation complexes in human cells. Mutations in mitochondrial DNA (mtDNA) have been associated with a wide variety of human disorders ranging from optic atrophy, deafness, diabetes to peripheral neuropathy or myopathy [1]. Most of the pathogenic mutations in human mtDNA are heteroplasmic (i.e. coexistence of mutant and wild-type genomes in a same cell) and their phenotypic expression is intimately linked to the ratio between mutant mtDNA molecules and wild-type ones (heteroplasmy level) [2]. This ratio can be variable in different tissues of the patient and even in different cells of the same tissue and can change with age [3]. Phenotypic expression of mtDNA mutations can be different for the various loads of the same mutation [4]. Typically, the biochemical defects and associated symptoms will only appear if the heteroplasmy level exceeds a given threshold generally comprised between 60% and 95% of mutant mtDNA, and subtle heteroplasmy changes can have dramatic effects on a patient’s phenotype. Thus, the downshift of heteroplasmy level could potentially provide a therapeutic strategy for the mitochondrial disorders, and several laboratories work for establishing methods for removing detrimental mtDNA sequences (rev. in [5]).

For instance, the anti-genomic strategy consists in the specific cleavage of mutant mtDNA by targeted specific endonucleases [6], zinc finger nuclease [7] or transcription activator-like effector nuclease (TALEN) [8, 9]. Limitations of this strategy consist in important off-target cleavage, which leads to elimination of more than 85% of mtDNA pool thus decreasing the therapeutic potential (as it was recently demonstrated by Gammage et al. [10]), and the challenge to engineer the protein which has to recognize specific DNA sequences. MitoTALENs specificity also depends on the sequence of the DNA target site and thus not all the point mutations can be discriminated from the wild-type sequences [10].

Another approach, so-called anti-replicative strategy, consists in targeting mitochondria with small molecules able to specifically anneal with mutant mtDNA and to interfere with its replication [11, 12]. Many organisms import non-coding cytosolic RNAs into the mitochondria (rev. in [13–15]). Our team has studied the molecular mechanisms of yeast tRNA^Lys^ import into yeast and human mitochondria [16, 17] and identified structural import determinants able to target oligonucleotides into mitochondria of human cells [18]. These mitochondrial RNA vectors had been applied to target anti-replicative oligonucleotides, designed to specifically anneal with mutant mtDNA, into human mitochondria. In these studies, RNA mitochondrial import induced the heteroplasmy shift in several cellular models: a) human cybrid cells and patient’s fibroblasts bearing a heteroplasmic point mutation in ND5 gene [19] and b) human cybrid cell line bearing 60% of mtDNA affected by a large deletion (nucleotides 8363–15438) underlying a case of frequent mitochondrial pathology, the Kearns Sayre Syndrome (KSS) [12].

**Fig 1:**
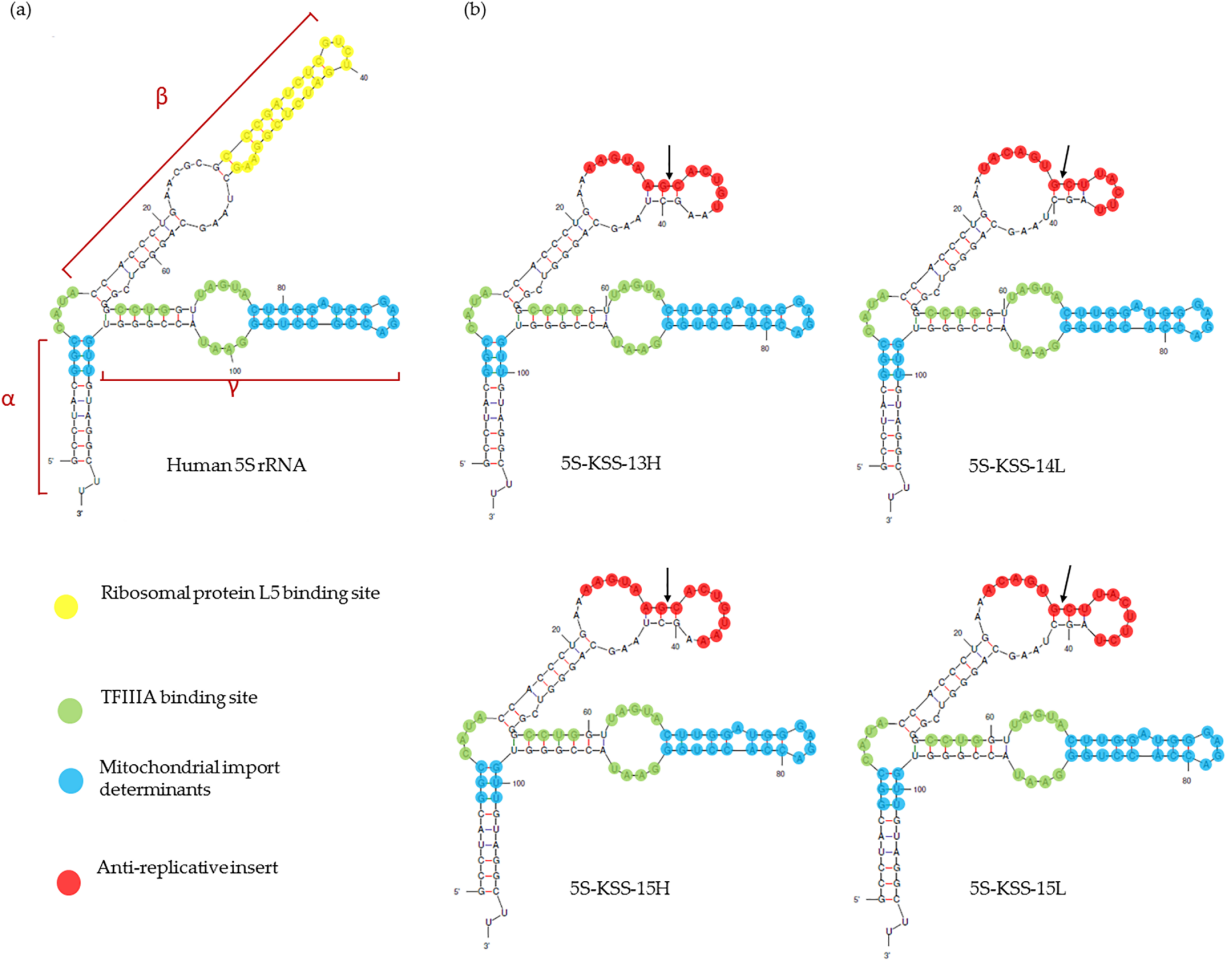
Secondary structure of wild type and recombinant 5S rRNAs. (a) Human 5S rRNA secondary structure, modified from [26]. (b) 2D structure of rec.5S rRNA versions, *mfold* predictions corrected manually. Sequences of the anti-replicative insertions (shown in red) correspond to boundaries of the KSS deletion in mtDNA; arrows indicate the deletion point.

Another type of RNA mitochondrial vectors is based on 5S rRNA expressed in nucleus and partially imported into human mitochondria [20–22]. 5S rRNA is a highly conserved and essential component of the large ribosomal subunit of all organisms, the only exceptions being mitochondrial ribosomes of yeast and mammals [23]. However, 5S rRNA seems to be the most abundantly imported RNA in mammalian mitochondria. Our studies demonstrated that 5S rRNA import mechanism relies on protein factors identified as the mitochondrial enzyme rhodanese and the precursor of mitochondrial ribosomal protein MRP-L18 [24, 25], interacting with two structural motifs located in the α and γ domains of 5S rRNA (Fig 1a), while a third motif corresponding to the distal part of the β domain and allowing the interaction with cytosolic ribosomal protein L5 may be either deleted or replaced without loss of import capacity [26]. This finding was exploited to demonstrate that 5S rRNA can function as a vector to deliver oligoribonucleotides into human mitochondria.

Our previous attempts to stably express 5S rRNA-based recombinant RNA (rec.5S rRNAs) in human cybrid cells resulted in a very weak expression level, estimated at merely 30±5 molecules per cell, though it allowed a moderate heteroplasmy shift. Moreover, variation in the rec.5S rRNAs expression obtained in different clones of transfected cells led to diverse effects on the mitochondrial heteroplasmy level (no shifts were detected in 70% of clones) [12].

In the present study, we aimed to increase the expression level of anti-replicative rec.5S rRNAs in human cybrid cells in a stable manner, and to verify if this expression can decrease the proportion of mutant mtDNA.

## Results

### Design of recombinant 5S rRNA molecules

To use 5S rRNA as a mitochondrial vector targeting mtDNA molecules affected by the KSS deletion, the distal portion of the β-domain of 5S rRNA (Fig 1a) was replaced by sequences corresponding to either H- or L-strand (standard annotation of two mtDNA strands, Heavy and Light ones) of the mtDNA at the junction of the KSS deletion boundaries [12].

**Fig 2:**
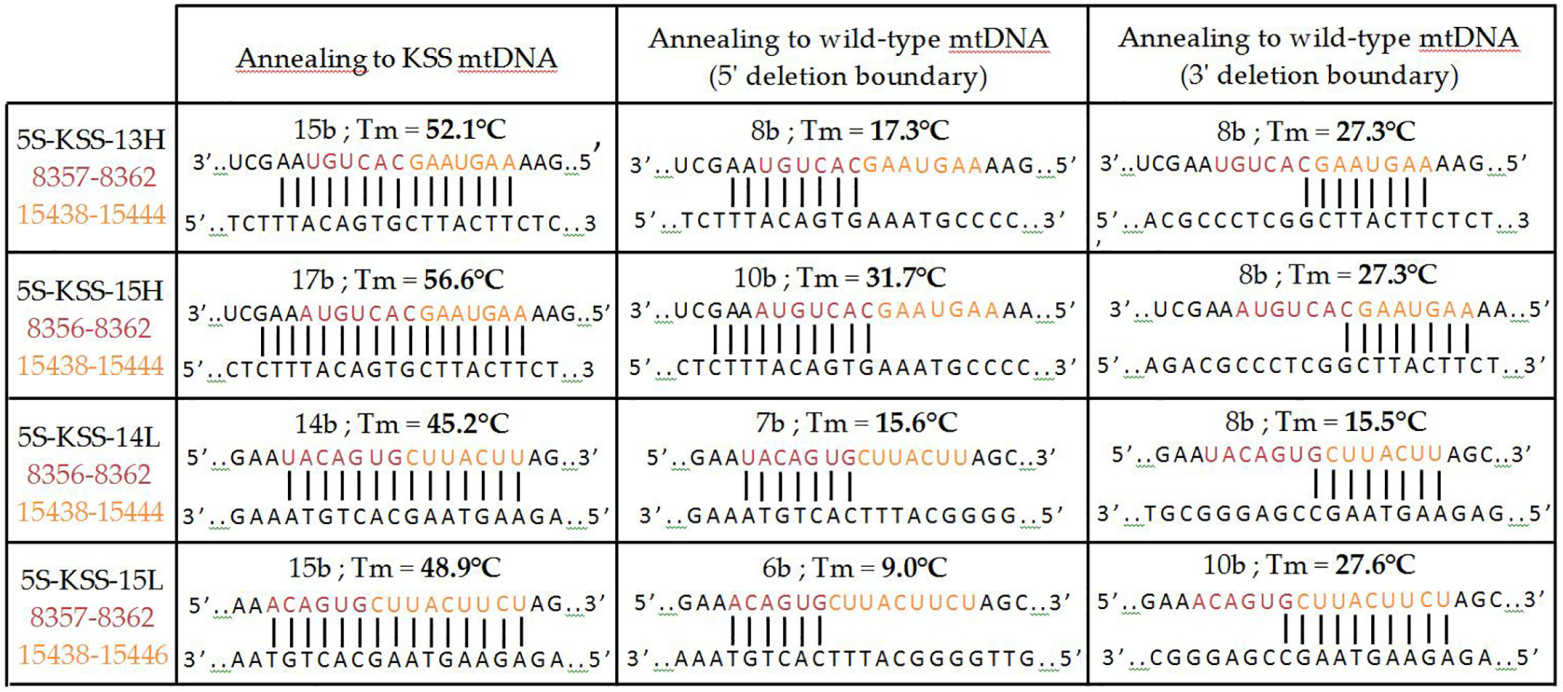
Structure and melting temperatures predictions for duplexes between rec.5S rRNA and mutant or wild-type mtDNA regions. Upper part of each duplex corresponds to the anti-replicative insertion of rec.5S rRNA indicated at the left. Nucleotides complementary to the 5’ boundary of the KSS deletion are shown in orange; those complementary to the 3’ deletion boundary are in green. 5S-KSS-13H and 5S-KSS-15H annealed to the L-strand of mtDNA; 5S-KSS-14L and 5S-KSS-15L annealed to the H-strand of mtDNA.

Previously, we designed two rec.5S rRNA versions bearing insertions corresponding to 13 nucleotides of the H-strand (5S-KSS-13H) or to 14 nucleotides of the L-strand (5S-KSS-14L) [12]. RNA 5S-KSS-13H was shown to be efficiently imported into isolated human mitochondria *in vitro* as well as *in vivo*, in cells transfected with corresponding RNA transcript [26]. In addition to these rec.5S rRNAs, we designed two new versions bearing insertions of 15 nucleotides (5S-KSS-15H and 5S-KSS-15L). For all these rec.5S rRNAs, the secondary structure predictions have been thoroughly analyzed (Fig 1b). All the versions were characterized by a classical 5S rRNA scaffold where the structure of the regions needed for interaction with transcription factor TFIIIA and the structural determinants of mitochondrial import have not been altered. *Cofold* analysis of the annealing between rec.5S rRNAs and the mutant mtDNA allowed prediction the length of the duplex region corresponding to 14 and 15 bp for 5S-KSS-14L and 5S-KSS-15L versions, and being slightly longer for 5S-KSS-13H and 5S-KSS-15H molecules (15 and 17 bp respectively, Fig 2). According to melting temperature predictions, Tm values are also different for rec.5S rRNAs targeting the L-strand of mtDNA (52.1°C and 56.6°C) and those targeting the H-strand (45.2°C and 48.9°C). Annealing of rec.5S rRNAs to wild-type mtDNA should be negligible at 37°C. This has been directly demonstrated by *in vitro* hybridizations of labeled rec.5S rRNAs with mtDNA fragments under physiological conditions (S1 Fig); the signal can be detected only for mutant mtDNA but not for the fragments of wild type mtDNA.

### Import of rec.5S rRNA molecules into human mitochondria

**Fig 3:**
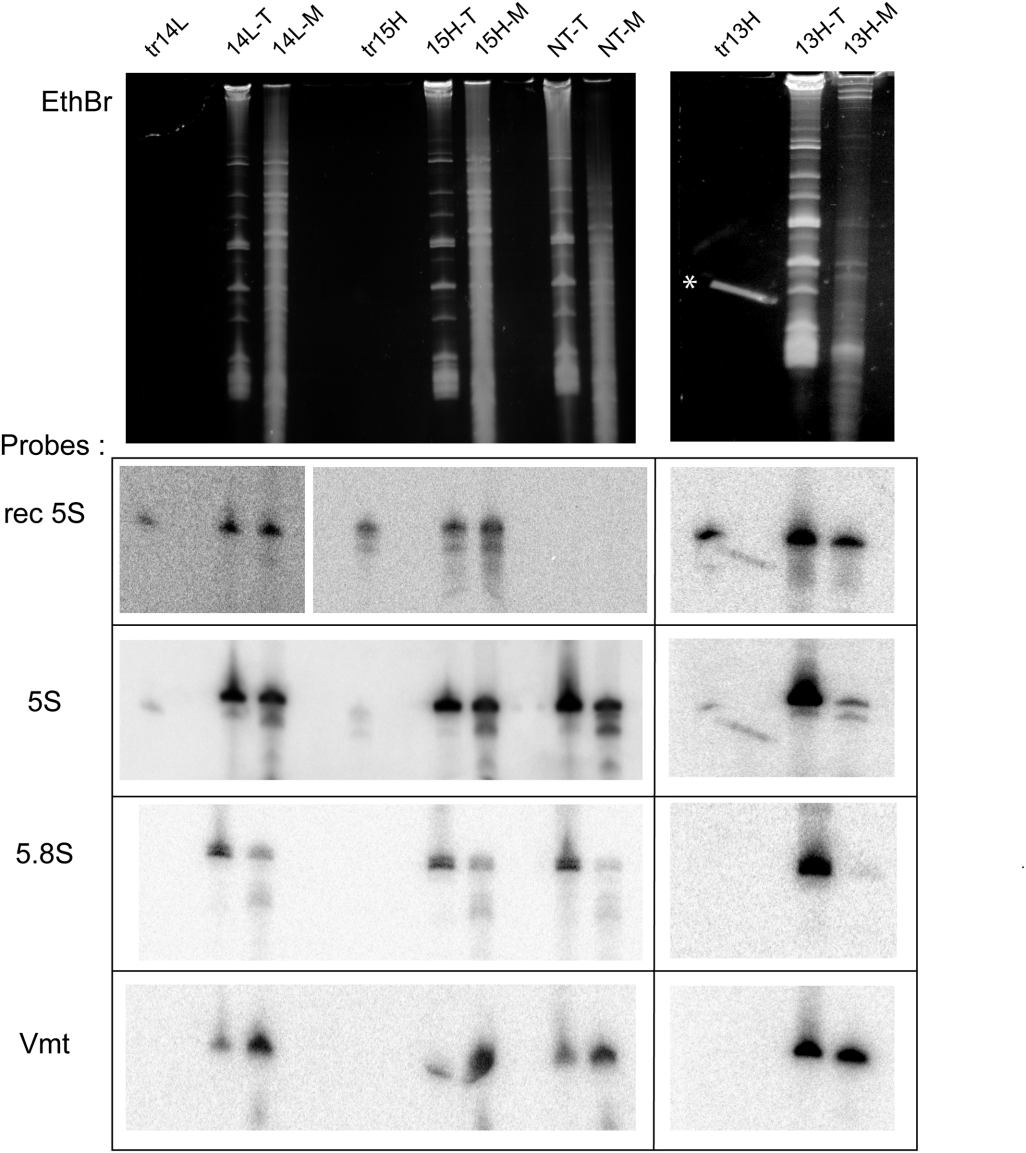
*In vivo* test of rec.5S rRNA import into human mitochondria. Northern blot analysis of rec.5S rRNA variants in total (T) and mitoplast (M) RNA preparations from cells transfected with various RNA, as indicated above the panels; NT, non-transfected cells; tr, 5ng of corresponding T7 transcript used for cell transfection. Gels stained with Ethidium bromide (EthBr) and hybridized with probes indicated at the left are shown. Rec 5S, probes corresponding to the insertion sequence specific for each rec.5S rRNA variant; 5.8S and Vmt, probes to cytosolic 5.8S rRNA and mitochondrial tRNA^Val^. 5S, probe to 5S rRNA, can hybridize with endogenous 5S rRNA and with rec.5S rRNA versions (which are 5–8 nucleotides shorter), thus giving double bands clearly visible in 13H-M sample. The long strip marked by * on the panel “tr13H, EthBr” is due to an artifact; 5ng of the transcript (tr13H) can be visible only by probing.

To evaluate the import efficiency of rec.5S rRNA variants *in vivo*, we transfected human cells with corresponding T7 transcripts as described previously [27]; RNA isolated from cells and from purified mitoplasts were analyzed by Northern blot hybridization (Fig 3 and S2 Fig).

Hybridization with a probe to mt tRNA^Val^ clearly demonstrates the enrichment of mitochondrial RNA transcript in mitoplasts preparations (this is not so visible for 5S-KSS-13H transfection, since a larger amount of total RNA was loaded on gel). Comparing to this enrichment, the levels of cytosolic contamination (probe to 5.8S rRNA) in mitoplasts fractions were quite negligible (<5%), therefore the samples can be used for quantification. To estimate the import efficiencies for various rec.5S rRNA, the ratio between the signals of the rec.5S rRNA in the mitoplast fraction and in total RNA preparation was normalized to that of the mitochondrial tRNA^Val^ in the same samples as described previously [26] (for details, see the Materials and methods section). Resulting import efficiencies obtained upon quantification of two transfection experiments were expressed as a percentage of the endogenous wild type 5S rRNA import efficiency (taken as 100%) estimated in control non-transfected cells (Table 1 and S2 Table).

**Table 1:** Relative efficiencies of rec.5S rRNA mitochondrial import.

5S rRNA	Relative import efficiency, % of endogenous 5S rRNA
Endogenous 5S rRNA	100%
5S-KSS-13H	200 ± 30
5S-KSS-14L	90 ± 5
5S-KSS-15L	70*
5S-KSS-15H	90 ± 15

* Data of one transfection experiment

The data demonstrate that all the rec.5S rRNA molecules can be imported into human mitochondria at the levels comparable with that of endogenous 5S rRNA. Surprisingly, only one version, 5S-KSS-13H, was characterized by improved import abilities. The reasons for this are uncertain, additional studies of folding and detailed structure of this RNA molecule can help to further improvement of the recombinant RNA import into mitochondria.

### Stable expression of rec.5S rRNA in human cybrid cells

Attempting to obtain an increased level of the stable expression of selected rec.5S rRNAs in human cybrid cells, we used the Flp recombinase-mediated integration of a gene of interest into specific genomic location by Flp-In^™^ T-Rex^™^ Core system (Invitrogen). To check the impact of rec.5S rRNA stable expression on the heteroplasmy level, we first had to introduce the FRT site into nuclear genome of the KSS cybrid cell line. These cells, obtained by the fusion of the patient’s cytoplasts with nuclei of immortalized osteosarcoma cells, contain a heteroplasmic population of mutant and wild type mtDNA molecules and were characterized by a small decrease of oxygen consumption [12]. Zeocin-resistant clones obtained after transfection with the pFRT/*lac*Zeo plasmid were characterized by similar growth rates and by a single integrated FRT site, as determined by Southern blot hybridization (S3 Fig).

Because integration of the pFRT/*lac*Zeo plasmid into the genome occurs randomly, expression levels of the *lacZ*-Zeocin fusion gene will be dependent on the transcriptional activity of the surrounding sequences at the integration site. Thus, Zeocin-resistant clones were screened for β-galactosidase activity levels. For all of them, specific β-gal activities were 4 to 8-fold lower compared to the commercial HEK 293 T-Rex^™^ Flp-In ^™^ cells (S3 Table). Clone 5, selected for further experiments (referred to as FRT-KSS cell line), was characterized by the highest β-gal activity and by 51±5% KSS heteroplasmy level.

**Fig 4:**
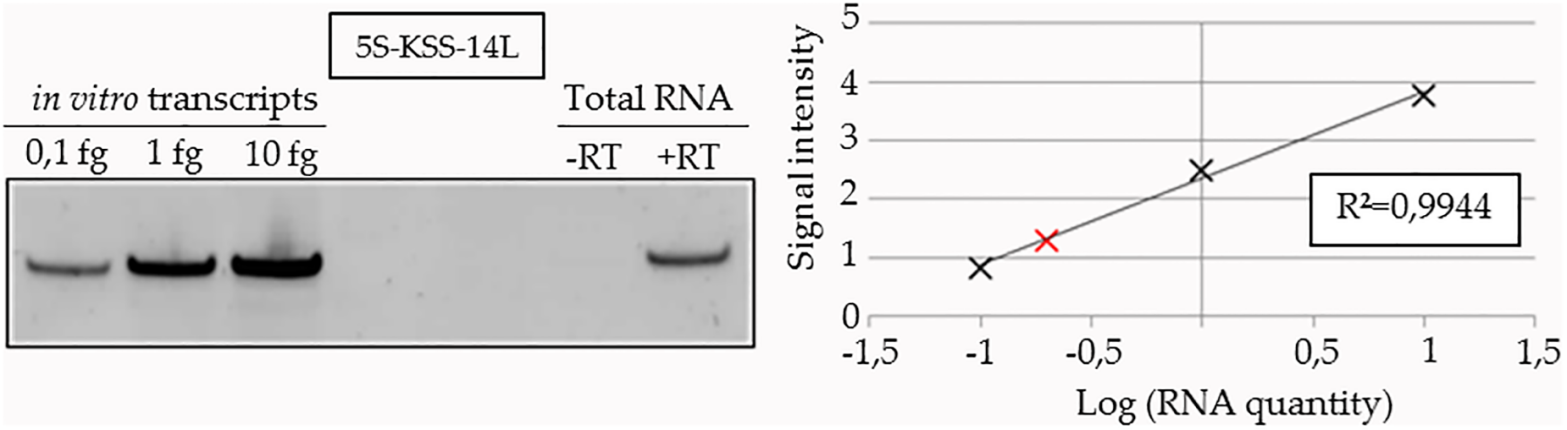
Quantification of 5S-KSS-14L RNA expression by semi-quantitative RT-PCR. On the left, PAGE analysis of the RT-PCR reactions performed on the total cellular RNA or known amounts of purified T7-transcripts, as indicated above the panel. On the right, an example of the calibration curve used for quantification of 5S-KSS-14L RNA (in red) in cybrid cell line.

Genes of rec.5S rRNA versions with natural flanking regions were cloned into pcDNA^™^5/FRT/TO vector and then integrated into FRT site of the nuclear genome of FRT-KSS cybrid cell line. Expression levels of rec.5S rRNAs have been assayed by semi-quantitative RT-PCR using corresponding T7-transcripts to create a calibration curve (Fig 4) and by real-time RT-qPCR (S4 Table).

We performed several independent transfections of FRT-KSS cells with pcDNA^™^5/FRT/TO plasmids. The first observation was that the expression of the transgenic rec.5S versions varied in different cell lines even though the FRT system is dedicated to provide a controlled level of expression directed from a given locus. Indeed, we generated lines either with high rec.5S rRNAs expression levels (referred for as 5S-KSS-13H^(1)^ and 5S-KSS-14L^(1)^), or those characterized by low expression for 5S-KSS-13H, 5S-KSS-14L and 5S-KSS-15L, or a medium expression level for 5S-KSS-15H (Table 2 and S4 Table). One may explain the observed variability in the expression level by multiple insertions of pcDNA^™^5/FRT/TO-13H and -14L plasmids into nuclear genome in the case of the best expressers 5S-KSS-13H^(1)^ and 5S-KSS-14L^(1)^, while single copy of rec.5S rRNA gene has been integrated in other transgenic lines. If this stands true, it can indicate that the rec.5S rRNAs expression level could be improved by integration of multiple copies of rec.5S rRNA gene in nuclear genome.

**Table 2:** Amounts of anti-replicative molecules in rec.5S rRNA expressing cell lines.

Rec.5S rRNA	Number of rec.5S rRNA/cell
5S-KSS-13H^**(1)**^	2000±200
5S-KSS-13H	200±30
5S-KSS-14L^(**1**)^	2000±200
5S-KSS-14L	300±50
5S-KSS-15H	1300±200
5S-KSS-15L	200±30

### Shift of the heteroplasmy levels in transgenic cell lines

**Fig 5:**
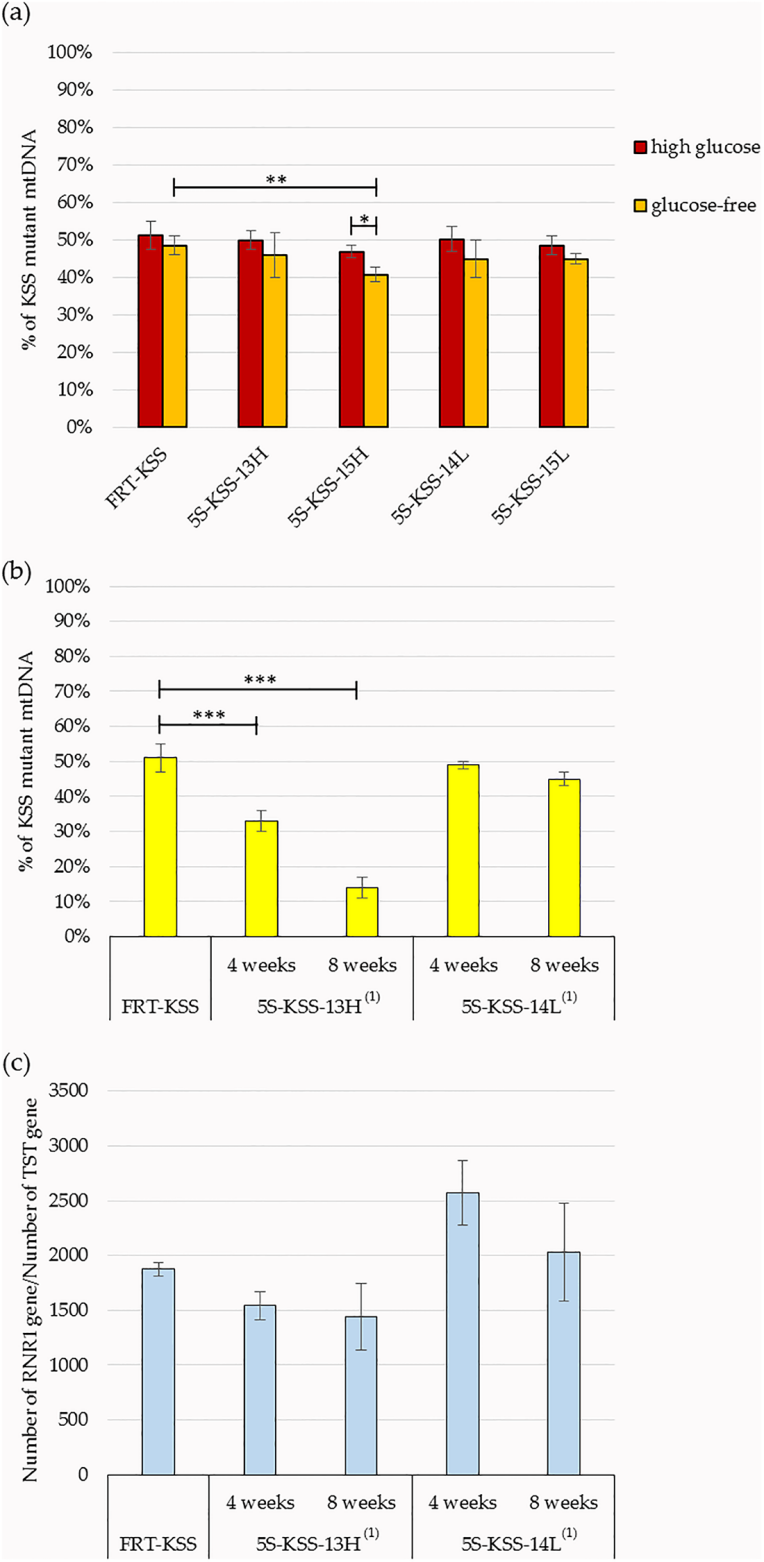
Effect of rec.5S rRNA expression on the heteroplasmy level of KSS mutation. (**a**) Proportion of KSS mtDNA (Y axis) in cybrids cell lines with low expression of rec.5S rRNA versions (see Table 1) after 8 weeks of cultivation in high glucose or glucose-free media, as indicated. (**b**) Time-depended decrease of KSS mutation load in cells expressing high amounts of rec.5S rRNAs 5S-KSS-13H^(**1**)^ and 5S-KSS-14L^(**1**)^ in glucose-free medium. (**c**) MtDNA copy numbers normalized to nuclear gene *TST1*, measured in the same cell populations as in (**b**). Cells issued from independent transfections were cultivated separately; each population was divided into 4 parallel cultures. Total DNA was isolated from a portion of each cell cultures every 2 weeks and analysed by 2–3 independent qPCR experiments, each measure performed in triplicates. Standard deviations are calculated from qPCR data obtained on the independently cultured cells (n = 4). Statistical differences were determined with a two-tailed Student’s *t*-test (*, p < 0.05; **, p < 0.01; ***, p < 0.001).

To check if the stable expression of rec.5S rRNA molecules can induce a mtDNA heteroplasmy shift in the human cybrid cells, we cultivated FRT-KSS cell lines for 8 weeks and measured the mutant mtDNA load by real-time qPCR as described previously [12]. Cells issued from independent transfections were cultivated separately, each one divided into 4 parallel cultures. Total DNA was isolated from a portion of cells every 2 weeks and analyzed by 2–3 independent qPCR experiments with each measure performed in triplicates. First, we compared the KSS heteroplasmy levels in the transgene and control cell lines after cultivation in a high glucose medium, but failed to detect any significant shift of the proportion between mutant and wild-type mtDNA molecules during 8 weeks cultivation (Fig 5a, “high glucose” samples).

To investigate if the selection for better mitochondrial respiration could create an advantage for cells with decreased heteroplasmy levels, we cultivated transgenic cells in a carbohydrate-free medium. In these conditions, only pyruvate and amino acids are available as carbon sources and cells should rely on mitochondrial oxidative phosphorylation to produce ATP.

Cybrid cells FRT-KSS were able to grow in a medium without addition of glucose and demonstrated only little heteroplasmy variations after 8 weeks of culture (Fig 5a) indicating that there were no significant random fluctuations in heteroplasmy. In all transfected cell lines with low expression of rec.5S rRNA versions, a very moderate decrease of the KSS heteroplasmy level has been observed in selective conditions. Statistically significant impact on the heteroplasmy has been detected only in 5S-KSS-15H expressing cells, where the mutation load was shifted from 51±4% to 41±2% (P-value<0.01) (Fig 5a). For this cell line, we obtained a tiny decrease (about 5% of heteroplasmy shift) after 4 weeks of cultivation (not shown), after 8 weeks we detected statistically significant decrease (10% of heteroplasmy shift), and this level didn’t change during further cultivation up to 4 months, and remained stable after freezing-thawing of the cells.

We next applied selective conditions to the cell lines with best expression of a rec.5S rRNA, namely 5S-KSS-13H^(1)^ and 5S-KSS-14L^(1)^. In the case of 5S-KSS-13H^(1)^, proportion of the mutant mitochondrial genomes has been significantly decreased in a clear time-dependent manner to reach 13±3% (Fig 5b). This level was thereafter stable during cell cultivation in a media either with or without glucose. Noteworthy, the 5S-KSS-13H RNA was characterized by 2 fold improved import into mitochondria (Table 1), which may explain, along with its high expression, the improved effect on the heteroplasmy load compared to the other rec.5S rRNA versions.

We suppose that the stable expression and mitochondrial import of the anti-replicative 5S rRNAs in cybrid cells can induce a shift of equilibrium between mutant and wild-type mitochondrial genomes. This shift can be more or less dramatic depending on the level of 5S rRNA expression. Thereafter, cells can adapt to a new equilibrium (may be by reprogramming of metabolic pathways, as it was demonstrated for MELAS mutation in [4]) and the obtained new heteroplasmy level remains stable during further cultivation.

On the other hand, high expression of the 5S-KSS-14L^(1)^ version did not induce an important decrease of heteroplasmy. Since two rec.5S rRNA versions, 13H and 14L, are designed to target different mtDNA strands, one can hypothesize that the different anti-replication action of these rec.5S rRNA molecules may be related to the asymmetric replication mechanism specific for mammalian mtDNA (see Discussion for details).

To ascertain the absence of a non-specific effect on replication that could reduce total mtDNA copy number in transfected cells, we estimated mtDNA amounts in FRT-KSS cybrid cell line before transfection, and in 5S-KSS-13H^(1)^ and 5S-KSS-14L^(1)^ cells after 4 and 8 weeks of cultivation in glucose-free medium (Fig 5c). Using real-time PCR, we measured a copy number of all mtDNA molecules (gene 12S rRNA) compared to nuclear DNA (gene of rhodanese *TST1*). We detected ≈20% decrease of mtDNA copy number in 5S-KSS-13H^(1)^ cell line. In theory, inhibition of the replication of 50% of mtDNA genomes (bearing KSS mutation) should cause a 25% decrease of mtDNAcn after one round of the replication. It is difficult to build a mathematical model for the action of anti-replicative molecules, since we do not know the velocity of mtDNA replication and exact level of affected mutant genomes. Still, some small level of mtDNA copy number downshift can be anticipated. Noteworthy, in case of an unspecific effect on mitochondrial replication process, we might expect a much more important depletion of mtDNA due to replication stalling of both, mutant and wild type mitochondrial genomes. Therefore, our results indicate that no depletion effect has been caused by an eventual off-target effect on the wild-type mtDNA replication.

Obtained new data indicate that the impact of anti-replicative rec.5S rRNA molecules on the mutant mtDNA load is strongly dependent on the amount of rec.5S rRNA molecules. High levels of RNA expression and mitochondrial import seem to be necessary to affect the mutant mtDNA replication and thus change the proportion between mutant and wild-type mitochondrial genomes.

## Discussion

### 5S rRNA as a mitochondrial RNA vector

Natural pathway of small non-coding RNA trafficking into mitochondria has been described in phylogenetic groups as diverse as protozoan, plants, fungi and animals [28, 29]. Small 5S ribosomal RNA is one of the most abundant cytosolic RNA imported into mitochondria. It had been reported that 5S rRNAs are expressed from large nuclear gene arrays (100–200 genes) and that a small portion of its cellular pool can readily be found within mitochondria in different organisms, from flies to humans [20, 21, 30]. In mammals, the mitochondrial function of 5S rRNA is not known. Cryo-electronic microscopy analysis recently demonstrated that the mitochondrial ribosomes from pig liver and human cultured cells do not contain 5S rRNA, but its place in the central protuberance of the large ribosomal subunit is occupied by mitochondrial tRNA^Phe^ or tRNA^Val^ correspondingly [31, 32]. The absence of the canonical function for 5S rRNA in human mitochondria raised some doubts concerning its mitochondrial import [33, 34]. This opinion, not supported by direct experimental data, does not make a distinction between two independent points: 1) mitochondrial localization of a small portion of the cytosolic 5S rRNA pool and 2) 5S rRNA integration into the large subunit of the mitochondrial ribosome. The absence of 5S rRNA in the large ribosomal subunit does not refute the ability of the mammalian organelle to import RNA from the cytoplasm. Moreover, the mitochondrial import of small amounts of RNA may indicate on a regulatory function of these molecules, for instance, 5S rRNA might participate in the assembly of mitochondrial ribosome (I. Tarassov, in preparation).

Even being rather inefficient compared to protein mitochondrial import, RNA targeting can be used to address into human mitochondria tRNA molecules replacing those affected by mtDNA mutation [18, 35] or the anti-replicative oligonucleotides with a therapeutic potential [12, 19, 36].

We previously demonstrated that 5S rRNA can function as a vector to deliver oligoribonucleotides into human mitochondria, and this principle has been successfully applied by three independent laboratories [30, 37, 38]. In the present study, we analyzed mitochondrial import of four 5S rRNA molecules bearing the 14–15 bases substitutions in the distal portion of the β-domain. We detected the presence of all the rec.5S rRNA versions in purified mitoplasts, and their import efficiencies were close to that of endogenous wild type 5S rRNA.

In contrast to small artificial anti-replicative RNAs used previously for the transient transfection of human cells [19], recombinant molecules based on 5S rRNA structure can be expressed in cells in a stable manner due to the internal promoter region recognized by RNA Polymerase III, then be processed, exported from the nucleus and imported into mitochondria. Nevertheless, the discrepancy between 100–200 copies of wild-type 5S rRNA gene and a unique gene of rec.5S rRNA per genome creates a competition between 5S rRNA molecules for the transcription, maturation and export protein factors. Thus, the high expression of rec.5S rRNA molecules in human cells should be a prerequisite of their anti-replicative activity.

### Expression of anti-replicative rec.5S rRNA molecules

In the present study, we used the Flp recombinase-mediated integration of a gene of interest into transcriptionally active genomic location. This approach allowed an improvement of rec.5S rRNAs expression compared to the random insertion of plasmid DNA bearing rec.5S rRNA genes [12]. Nevertheless, a significant heteroplasmy shift has been detected in cells bearing only one version of rec.5S rRNA genes, namely 5S-KSS-13H, expressed at the high level (Table 2). Lower expression of the same molecule did not affect mutant mtDNA load, indicating on an anti-replicative RNA concentration threshold. Surprisingly, 5S-KSS-15H version, which differs by 2 bp longer duplex predicted with mutant mtDNA, provided only a small heteroplasmy decrease. This can be explained by a slightly lower expression and lower mitochondrial import of this RNA. Another difference between these constructs consists in direct orientation of 5S-KSS-15H gene in respect to CMV promoter in the pcDNA^™^5/FRT/TO cloning vector, in contrast with the inverse orientation of 5S-KSS-13H gene. Thus, we cannot exclude possible synthesis of a longer 5S-KSS-15H transcript from external CMV promoter, which could be detected by RT-PCR therefore compromising the full-size 5S-KSS-15H RNA quantification. These considerations should be taken into account in the further studies aiming to increase the expression of anti-replicative RNA molecules in a more reproducible way by using constructs bearing only promoters for RNA polymerase III.

The mechanism of human mtDNA replication is still a subject of intense debate (rev. in [39]). The idea of anti-replicative heteroplasmy shift as a therapeutic strategy [11] was initially based on the strand-displacement mtDNA replication model [40] that suggested the existence of long single-stranded replication intermediates (S4 Fig). Discovery of RNA-DNA hybrid intermediates gave rise to RITOLS model (Ribonucleotide Incorporation Throughout the Lagging Strand) [41]. A third model proposed conventional strand-coupled DNA synthesis, initiating from sites dispersed across the broad zone named ori Z [42]. Apparently, all the three mtDNA replication mechanisms can take place in mitochondria, depending on the growth conditions and the energetic state of the cells. For instance, recent analysis of mtDNA replication in mice tissues had shown that liver and kidney cells use the asynchronous mechanism, while heart, brain and skeletal muscle employ a strand-coupled replication mode [43].

Previously, we have directly demonstrated mtDNA replication stalling induced by small anti-replicative RNA, detected as a new replication intermediate on 2D agarose gel [12]. This stalling, occurred at the site of the RNA annealing due to impairing of the replication fork progression, which can be suggested for all the three current models of mtDNA replication (S4 Fig), since the replisome helicase is unable to displace RNA from the short RNA-DNA hybrids [42]. Our present data indicate that anti-replicative RNAs 5S-KSS-13H and 5S-KSS-15H, bearing insertions complementary to mtDNA L-strand, are able to induce a downshift of the KSS mutation load. Expression of two other rec.5S rRNA versions, 5S-KSS-14L and 5S-KSS-15L, designed to target the H-strand of mtDNA, had no significant impact on the KSS heteroplasmy level. This can be related to asymmetric strand-displacement mechanism of human mtDNA replication, supposing that at the first step, only L-strand is used as a template for DNA synthesis [40], therefore, its targeting by anti-replicative RNAs 5S-KSS-13H and 5S-KSS-15H, as shown schematically on S4 Fig, should be more efficient compared to the H-strand targeting RNAs. This is relevant to recently published data providing a strong support of the strand-displacement model of mtDNA replication in cultured cells [44].

One should also take into account that melting temperatures predicted for rec.5S rRNA-mtDNA duplexes are lower for 5S-KSS-14L and 5S-KSS-15L versions (45.2°C and 48.9°C) compared to 5S-KSS-13H and 5S-KSS-15H ones (52.1°C and 56.6°C, Fig 2). This can provide another reason for the poor anti-replicative abilities of 5S-KSS-L versions. Noteworthy, a very recent paper from the lab of Pierre Rustin [45] provided a set of evidences that human mitochondria are maintained at close to 50°C if the respiratory chain is functional. We can hypothesize that in fully active mitochondria, 5S-KSS-14L and 5S-KSS-15L RNAs cannot anneal to mtDNA due to increased temperature in mitochondrial matrix.

All these considerations will help to optimize the anti-replicative RNA structure and to design rec.5S rRNA molecules targeting not only mtDNA deletions, but also pathogenic point mutations, as it was done for small artificial RNAs [12].

### Selective cell growth conditions improved the anti-replicative RNA effect

Warburg and Crabtree effects describe the ability of rapidly proliferating cells to favor glycolysis. Recent studies suggest that cells can adapt to mitochondrial dysfunction by switching to glycolysis, despite aerobic conditions [46]. Development of *trans*mitochondrial cybrids by fusion of enucleated somatic cells harboring pathological mtDNA mutations with a cell line depleted of their mtDNA has enabled the study of consequences of mtDNA mutations [47]. However, cybrid cells cultured in media with high concentrations of glucose tend to acquire highly glycolytic phenotypes, which make them less suitable as models for studying mitochondrial dysfunction.

Attempts have been made to overcome this phenomenon, by substituting glucose with galactose, which does not support anaerobic glycolysis [48]. This is usually explained by the fact that galactose cannot be oxidized to pyruvate without prior conversion to glucose, which consumes two molecules of ATP, thus making anaerobic glycolysis useless as a source of energy [49]. Nevertheless, some cell lines cannot utilize galactose, but can grow in a carbohydrate-free media, apparently relying on the metabolism of pyruvate and amino acids, thus galactose-containing media can be replaced by carbohydrate-free ones. It was demonstrated that the activity of all respiratory chain complexes tended to be higher in the glucose-starved cells [50], since they are forced to rely on mitochondrial oxidative phosphorylation to meet their energy requirements.

Previously, few studies addressed the question of metabolically induced mtDNA heteroplasmy shifting. The ketogenic medium has been shown to shift the heteroplasmy level of cybrid cells towards the wild type [51]. Another report demonstrated that the apparently homoplasmic 100% MELAS cybrid cells kept in low-glucose medium have shifted their heteroplasmy level to 90% [52]. However, a more recent publication from the same research team demonstrated that the improvement of mitochondrial function in MELAS cybrid cells exposed to a diet combining low glucose and ketone bodies was not connected to a heteroplasmy shift, but to increase of the mtDNA copy number [53], therefore, the selective pressure alone was not capable to induce the heteroplasmy shift.

Noteworthy, in our experiments, shifts in mutant mtDNA proportion have been detected only for cells cultivated in a medium poor in glucose. These data are in accordance with the hypothesis that culturing mutant cells in a low-glucose medium and thus forcing them to use oxidative phosphorylation would create a selective advantage for cells with improved respiration capacities.

## Conclusions

In the present study, we attempted to induce the heteroplasmy shifting by stable expression of anti-replicative 5S rRNA molecules capable to slow the mutant mtDNA replication in human *trans*mitochondrial cybrid cells and thus decreasing proportion between mutant and wild type mtDNA molecules. We provide the strategy of anti-replicative rec.5S rRNA selection based on the melting temperature prediction and analysis of mitochondrial import. The heteroplasmy shift, dependent on the level of the recombinant 5S rRNA expression and import, can be modulated by cell growth conditions. Applying selective conditions favorable for the cells where the mutant mtDNA proportion has been successfully decreased, allowed detection of the change in heteroplasmy load for cybrid cells bearing a large deletion in mtDNA. In contrast to recently developed engineered nuclease technology, which induced a substantial off-target effects and a significant depletion of mtDNA [10], we detected only a small decrease of mtDNA copy number, which can be caused by the stalling of the mutant KSS genomes replication. This indicates that the decreased proportion between mutant and wild type mtDNA molecules is not a consequence of a random repopulation of depleted pool of mtDNA genomes, but can be induced by the anti-replicative 5S rRNA expression.

Considering the possible therapeutic applications, the advantage of the stable rec.5S rRNA expression consists in non-reversible heteroplasmy shift, in contrast with a transient KSS mutation load decrease obtained previously by use of small anti-replicative RNAs [12]. Nevertheless, we detected the obvious limitations of the approach used: need of high level of rec.5S rRNA expression and an important variability of this level in transfected cells, influencing the impact on the heteroplasmy. Further development of the strategy can be achieved by integration of multiple copies of anti-replicative rec.5S rRNA molecules expressed from external PolIII promoter by use of plasmid or viral vectors.

In conclusion, our data demonstrate that expression of anti-replicative molecules based on 5S rRNA structure in human cybrid cells can induce a stable shift in a heteroplasmy level of pathogenic mutations in mtDNA. This shift, dependent on the rec.5S rRNA sequence and the level of its expression, can be modulated by cell growth conditions, thus opening possibility to improve models of gene therapy exploiting the anti-replicative strategy.

## Materials and methods

### Human cell lines and culture conditions

Human *trans*mitochondrial cybrid cells containing 65±2% of mtDNA molecules affected by KSS deletion (nucleotides 8363–15438) obtained by the team of Dr A. Lombes, Inst. Cochin, Paris [12] were cultivated in DMEM medium (Sigma) containing 4.5 g/L glucose, 0.584 g/L L-glutamine and supplemented with 50 mg/L uridine and 3.7 g/L sodium bicarbonate (for glucose-rich conditions). For glucose-free conditions, DMEM base (Sigma) was supplemented with 50 mg/L uridine, 3.7 g/L sodium bicarbonate, 0.584 g/L L-glutamine and 108 mg/L sodium pyruvate. Cybrid cells transfected with pFRT/*lacZeo* were cultivated in media containing 100 μg/mL Zeocin (Invitrogen). Cells transfected with pcDNA^™^5/FRT/TO were cultivated in media supplemented with 150μg/mL Hygromycin B-Gold (InvivoGen).

### Design and synthesis of recombinant 5S rRNA molecules

Secondary structures of rec.5S rRNA were predicted using the *Mfold* software [54] and corrected in accordance with data on human 5S rRNA structure [55]. Analysis of the annealing between rec.5S rRNAs and the mtDNA was performed by *RNAcofold web server*, University of Vienna. To estimate melting temperatures for RNA-DNA duplexes, we used IDT Sci-Tools *OligoAnalyzer 3*.*1* software [56].

Rec.5S rRNAs were obtained by T7 transcription using the T7 RiboMAX Express Large-Scale RNA Production System (Promega) and gel-purified. To create PCR templates, we used a two-step protocol: PCR1 was performed on a plasmid bearing the human 5S rRNA sequence under the control of the T7 promoter [26] using forward primer n°1 and reverse primers n°2 to 5 (S1 Table). Resulting PCR1 product was used as forward primer for PCR2 performed on the same plasmid template with reverse primer n°6 creating a DNA fragment containing rec.5S RNA sequence under the control of the T7 promoter. Plasmid or PCR fragment were cleaved by BglII (Fast Digest, ThermoScientific) to assure formation of the exact 5S rRNA 3’ end.

### Rec.5S rRNA *in vitro* hybridization and *in vivo* import test

To test specific rec.5S rRNA annealing with target mtDNA, wild-type or mutant mtDNA fragments (nucleotides 15,251–15,680 of wild-type mtDNA or 8,099–8,365/15,438–15,680 of mutant mtDNA) were amplified as described previously [12] using primers n°7 and 8 or n°8 and 9 respectively, separated on 1% agarose gel, blotted to Amersham Hybond-N membrane (GE Healthcare) and hybridized with ^32^P-labeled recombinant RNA in 1X PBS at 37 °C. Hybridization signals were revealed by Typhoon Trio (GE Healthcare).

The *in vivo* import assay on the cells transfected with rec.5S rRNA molecules was performed as described previously [17, 26, 27]. Briefly, 40h post transfection, cells were disrupted, mitochondria were isolated by differential centrifugation, treated with RNase A (Sigma) 10 μg/ml for 10 min at 25°C, washed three times, then treated with of 0.1% digitonin (Sigma) solution for 10 min at 25°C to disrupt the mitochondrial outer membrane. The mitoplast pellet was washed and resuspended in TRIZol reagent (Invitrogen). RNA isolated from the mitoplasts and from an aliquot of the transfected cells were separated by 8% urea-PAGE and analysed by Northern blot hybridization with ^32^P-labeled oligonucleotide probes (S1 Table, n°25–29). To avoid the discrepancies caused by the loading of different amounts of material, we used the hybridization signals corresponding to the mitochondrial tRNA^Val^ as a loading control. Thus, we take into account not the absolute intensity of hybridization signals but the ratio between the signals corresponding to rec.5S rRNAs and the host mitochondrial valine tRNA either in mitoplast RNA preparations (Ratio I) or in the total cellular RNA (Ratio II). Import efficiency of each rec.5S rRNA was calculated as a quadruple ratio between Ratios I and II and expressed in the form of percentage of the efficiency thus obtained for the endogenous wild type 5S rRNA in non-transfected cells.

$$Import\;efficiency=\frac{RI}{RII}$$

$$RI\;(mitoplast\;RNA)=\frac{rec.5S\;rRNA\;}{mt\;tRNA\;Val}$$

$$RII\;(total\;RNA)=\frac{rec.5S\;rRNA\;}{mt\;tRNA\;Val}$$

Cytosolic contamination was checked by hybridization with a probe to 5.8S rRNA, a component of cytosolic ribosomes. The levels of cytosolic contamination were subtracted from the import efficiency values for each rec.5S rRNA version (S2 Table). In control experiments, mitochondria were lysed before RNase treatments to demonstrate that all the RNAs were completely degraded (not shown).

### Production of a human transmitochondrial cybrid cell line bearing FRT site

To generate a KSS cybrid cell line bearing an FRT site, we used the Flp-In^™^ T-Rex^™^ Core system (Invitrogen). Cybrid cells were transfected with the pFRT/*lacZeo* plasmid according to the manufacturer’s protocol, and Zeocin-resistant clones were isolated.

To determine the number of FRT sites in each zeocin-resistant clone, genomic DNA was extracted, digested with HindIII (ThermoScientific), separated on a 1% agarose gel and blotted to Amersham Hybond-N membrane (GE Healthcare). To detect FRT sites, a fragment of the *lacZ* gene was amplified according to the manufacturer’s protocol (Invitrogen) and ^32^P-labelled using Prime-a-gene Labelling System (Promega). After hybridization at 65°C and washing, signals were revealed by phosphorimaging in Typhon Trio (GE Healthcare).

Specific β-galactosidase activity in cellular lysate were determined for each clone by use of the β-gal assay kit (Invitrogen) following manufacturer’s instructions and quantified as nmol of hydrolyzed substrate ONPG/30 min/mg of cellular protein.

### Generation of transmitochondrial cybrid cell lines expressing rec.5S rRNA

Rec.5S rRNA genes were obtained by a two-step protocol: 1) PCR1 with partially overlapping primers: forward primers n°10 (for 5S-KSS-15H and 5S-KSS-15L) or n°12 (for 5S-KSS-13H and 5S-KSS-14L) and reverse primers n°2 to 5. 2) Resulting DNA fragments were used as forward primers for PCR2 with reverse primer n°11 (for 5S-KSS-15H and 5S-KSS-15L) or n°13 (for 5S-KSS-13H and 5S-KSS-14L) and a plasmid containing human 5S rRNA gene with flanking regions [26] as a template.

Then, rec.5S rRNA genes were cloned into the pcDNA^™^5/FRT/TO plasmid vector (Invitrogen) and verified by sequence analysis. Cybrid FRT-KSS cells were co-transfected with pcDNA^™^5/FRT/TO-rec.5S rRNA constructs and pOG44 plasmid encoding Flp recombinase (Invitrogen) at a molar ratio 1:10 using Lipofectamine 2000 (Invitrogen). Forty-eight hours after transfection, Hygromycin B-gold (150μg/mL) (Invitrogen) was added to the medium for selection.

### Rec.5S rRNA quantification

Total cellular RNA was isolated with TRIZol reagent (Invitrogen). Levels of rec.5S rRNA expression were assayed by semi-quantitative RT-PCR with forward primers specific to the anti-replicative insert (primers n°18 to 21) (S1 Table) and reverse primer corresponding to the 3’-end of 5S rRNA (primer n°22) using One-step RT-PCR kit (QIAGEN). For each recombinant RNA, serial dilutions of the corresponding T7-transcript were used to create a calibration curve and thus estimate the amount of rec.5S rRNA molecules per cell. Control no-RT reactions were performed for each RNA sample. RT-PCR products were PAGE separated, visualized by ethidium bromide staining and quantified using G-box Software. This approach provided reproducible data, SD = 15–20%.

RT-qPCR was performed in two steps, first by reverse transcriptase Revertaid H minus (Thermofisher) with primer n°22, second by real-time qPCR kit Sso Advanced Universal SYBR Green Supermix (Biorad), primers n°18 to 21 (S1 Table), in triplicates, with no-RT control reactions. Amplification cycles were performed with C1000 Touch, CFX96^**™**^ Real-Time Detection System thermocycler (BioRad). PCR was performed by initial denaturation at 95°C for 10 min, followed by 40 cycles of 10s at 95°C and 45s at 55°C. The threshold cycle (Ct) values of each sample were used in the post-PCR data analysis by BioRad CFX Manager^**™**^ software 3.0. Absolute amounts of cDNA in each reaction were estimated by use of calibration curves made with corresponding plasmid DNA.

### Analysis of the heteroplasmy level and mtDNA copy number

Cells issued from independent transfections were cultivated separately, in 4 parallel cultures each one, total DNA was isolated from a portion of cells every 2 weeks and analyzed by 2–3 independent qPCR measurements in triplicates.

To isolate total DNA, cells were solubilized in 0.5 ml of buffer containing 10mM Tris–HCl pH 7.5, 10mM NaCl, 25mM Na-EDTA and 1% SDS. Then, 10 μl of proteinase K solution (20 mg/ml) was added and the mixture was incubated for 2 h at 50°. Finally, 50 μl of 5M NaCl was added and DNA was precipitated by isopropanol.

Heteroplasmy level for KSS deletion was analyzed by real-time qPCR using SYBR Green (C1000 Touch, CFX96^™^ Real-Time Detection System, BioRad) as described previously [12]. This approach allows quantification of the level of KSS deletion within a sample by comparing specific amplification of two different mtDNA regions, located within and outside of the deletion, in separate reactions. To improve the accuracy of the test, we estimated absolute amounts of DNA in each reaction by use of calibration curves and thus we calculated the ratio between all mtDNA molecules (mutant and wild-type) and the wild-type ones. Differences in KSS deletion level less than 5% were not considered as significant.

Primers are listed in the S1 Table. Two pairs of primers were used: 1) n°14 and 15 amplifying a 210bp fragment of 12S rRNA gene (nucleotides 1095–1305 in mtDNA) not affected by the KSS deletion as a value showing all mtDNA molecules, and 2) n°16 and 17 amplifying a 164 bp fragment in the deleted region (nucleotides 11 614–11 778) as a value showing only wild-type mtDNA molecules. Nuclear DNA quantification was performed using primers to rhodanese gene *TST1* (S1 Table). All reactions were performed in a 20 μl volume in triplicates. PCR was performed by initial denaturation at 95°C for 10 min, followed by 40 cycles of 30s at 95°C and 30s at 60°C. The threshold cycle (Ct) values of each sample were used in the post-PCR data analysis by BioRad CFX Manager^™^ software 3.0. In each experiment, absolute amount of DNA templates were determined using serial dilutions of linearized plasmid DNA bearing a corresponding sequence. The KSS heteroplasmy level was calculated using the formula: mutant mtDNA/total mtDNA = 1 − (WT mtDNA/total mtDNA). Data obtained on independently cultivated cells (n = 4) by 3–4 independent qPCR measurements were statistically processed using the two-tailed Student’s *t*-test; values of *p* ≤ 0.05 (*), *p* ≤ 0.001 (***) were considered to be statistically significant.

## Supporting information

S1 Fig Specific annealing of rec.5S rRNAs to mutant KSS mtDNA. Southern hybridization of wild-type (WT) or KSS mtDNA fragments (Mut) with ^32^P-labelled rec.5S rRNAs “5S-KSS-15H” and “5S-KSS-15L” (as indicated at the right) at 37°C in 1xPBS.
(TIF)

S2 Fig *In vivo* test of rec.5S rRNA import into human mitochondria. Northern blot analysis of rec.5S rRNA variants in total and mitoplast RNA preparations from cells transfected with various RNA (as indicated above the panels). Originals gel stained with Ethidium bromide (EthBr) and hybridized with probes indicated at the left, as on Fig 3.
(DOCX)

S3 Fig Analysis of the FRT site copy number in isolated *trans*mitochondrial cybrid KSS-FRT cell lines. Southern blot hybridization of genomic DNA from three KSS-FRT clones (2, 5 and 8) compared to commercial HEK 293 T-Rex^™^ Flp-In^™^ cells.
(TIF)

S4 Fig Schematic representation of three mtDNA replication models and possible effect of anti-replicative rec.5S rRNA (modified from [12]). (TIF)

S1 Table List of oligonucleotide primers. (DOCX)

S2 Table Quantification of mitochondrial import efficiencies of rec.5S rRNA. (DOCX)

S3 Table Characteristics of three isolated transmitochondrial cybrid KSS-FRT cell lines compared to commercial HEK 293 T-RexTM Flp-InTM. (DOCX)

S4 Table Quantification of anti-replicative molecules in rec.5S rRNA expressing cell lines by two approaches: Semi-quantitative one step RT-PCR and two steps real-time RT-qPCR. (DOCX)

## References

[1] Gorman GS, Schaefer AM, Ng Y, Gomez N, Blakely EL, Alston CL, et al Prevalence of nuclear and mitochondrial DNA mutations related to adult mitochondrial disease. Ann Neurol. 2015;77(5):753–9. Epub 2015/02/06. doi: 10.1002/ana.24362 . 25652200 10.1002/ana.24362 PMC4737121

[2] Wallace DC. Mitochondrial DNA mutations in disease and aging. Environ Mol Mutagen. 2010;51(5):440–50. doi: 10.1002/em.20586 . 20544884 10.1002/em.20586

[3] Ozawa M, Nonaka I, Goto Y. Single muscle fiber analysis in patients with 3243 mutation in mitochondrial DNA: comparison with the phenotype and the proportion of mutant genome. Journal of the neurological sciences. 1998;159(2):170–5. . 9741403 10.1016/s0022-510x(98)00152-x

[4] Picard M, Zhang J, Hancock S, Derbeneva O, Golhar R, Golik P, et al Progressive increase in mtDNA 3243A>G heteroplasmy causes abrupt transcriptional reprogramming. Proceedings of the National Academy of Sciences of the United States of America. 2014;111(38):E4033–42. doi: 10.1073/pnas.1414028111 . 25192935 10.1073/pnas.1414028111 PMC4183335

[5] Patananan AN, Wu TH, Chiou PY, Teitell MA. Modifying the Mitochondrial Genome. Cell metabolism. 2016;23(5):785–96. doi: 10.1016/j.cmet.2016.04.004 . 27166943 10.1016/j.cmet.2016.04.004 PMC4864607

[6] Alexeyev MF, Venediktova N, Pastukh V, Shokolenko I, Bonilla G, Wilson GL. Selective elimination of mutant mitochondrial genomes as therapeutic strategy for the treatment of NARP and MILS syndromes. Gene Ther. 2008;15(7):516–23. doi: 10.1038/sj.gt.2008.11 . 18256697 10.1038/gt.2008.11 PMC10416612

[7] Gammage PA, Rorbach J, Vincent AI, Rebar EJ, Minczuk M. Mitochondrially targeted ZFNs for selective degradation of pathogenic mitochondrial genomes bearing large-scale deletions or point mutations. EMBO molecular medicine. 2014;6(4):458–66. doi: 10.1002/emmm.201303672 . 24567072 10.1002/emmm.201303672 PMC3992073

[8] Bacman SR, Williams SL, Pinto M, Peralta S, Moraes CT. Specific elimination of mutant mitochondrial genomes in patient-derived cells by mitoTALENs. Nat Med. 2012;19(9):1111–3. doi: 10.1038/nm.3261 . 23913125 10.1038/nm.3261 PMC4153471

[9] Reddy P, Ocampo A, Suzuki K, Luo J, Bacman SR, Williams SL, et al Selective elimination of mitochondrial mutations in the germline by genome editing. Cell. 2015;161(3):459–69. doi: 10.1016/j.cell.2015.03.051 . 25910206 10.1016/j.cell.2015.03.051 PMC4505837

[10] Gammage PA, Gaude E, Van Haute L, Rebelo-Guiomar P, Jackson CB, Rorbach J, et al Near-complete elimination of mutant mtDNA by iterative or dynamic dose-controlled treatment with mtZFNs. Nucleic Acids Res. 2016;44(16):7804–16. doi: 10.1093/nar/gkw676 . 27466392 10.1093/nar/gkw676 PMC5027515

[11] Taylor RW, Chinnery PF, Turnbull DM, Lightowlers RN. Selective inhibition of mutant human mitochondrial DNA replication in vitro by peptide nucleic acids. Nat Genet. 1997;15(2):212–5. doi: 10.1038/ng0297-212 . 9020853 10.1038/ng0297-212

[12] Comte C, Tonin Y, Heckel-Mager AM, Boucheham A, Smirnov A, Aure K, et al Mitochondrial targeting of recombinant RNAs modulates the level of a heteroplasmic mutation in human mitochondrial DNA associated with Kearns Sayre Syndrome. Nucleic Acids Res. 2013;41(1):418–33. doi: 10.1093/nar/gks965 . 23087375 10.1093/nar/gks965 PMC3592399

[13] Tarassov I, Chicherin I, Tonin Y, Smirnov A, Kamenski P, Entelis N. Mitocondrial Targeting of RNA and Mitochondrial Translation In: Duchene AM, editor. Translation in Mitochondria and Other Organels: Springer Heidelberg; 2013 p. 85–109.

[14] Salinas-Giege T, Giege R, Giege P. tRNA biology in mitochondria. International journal of molecular sciences. 2015;16(3):4518–59. doi: 10.3390/ijms16034518 . 25734984 10.3390/ijms16034518 PMC4394434

[15] Kim KM, Noh JH, Abdelmohsen K, Gorospe M. Mitochondrial noncoding RNA transport. BMB reports. 2017;50(4):164–74. doi: 10.5483/BMBRep.2017.50.4.013 . 28115039 10.5483/BMBRep.2017.50.4.013 PMC5437960

[16] Kolesnikova OA, Entelis NS, Jacquin-Becker C, Goltzene F, Chrzanowska-Lightowlers ZM, Lightowlers RN, et al Nuclear DNA-encoded tRNAs targeted into mitochondria can rescue a mitochondrial DNA mutation associated with the MERRF syndrome in cultured human cells. Human molecular genetics. 2004;13(20):2519–34. doi: 10.1093/hmg/ddh267 . 15317755 10.1093/hmg/ddh267

[17] Gowher A, Smirnov A, Tarassov I, Entelis N. Induced tRNA import into human mitochondria: implication of a host aminoacyl-tRNA-synthetase. PloS one. 2013;8(6):e66228 doi: 10.1371/journal.pone.0066228 . 23799079 10.1371/journal.pone.0066228 PMC3683045

[18] Kolesnikova O, Kazakova H, Comte C, Steinberg S, Kamenski P, Martin RP, et al Selection of RNA aptamers imported into yeast and human mitochondria. Rna. 2011;16(5):926–41. doi: 10.1261/rna.1914110 . 20348443 10.1261/rna.1914110 PMC2856887

[19] Tonin Y, Heckel AM, Vysokikh M, Dovydenko I, Meschaninova M, Rotig A, et al Modeling of antigenomic therapy of mitochondrial diseases by mitochondrially addressed RNA targeting a pathogenic point mutation in mitochondrial DNA. J Biol Chem. 2014;289(19):13323–34. doi: 10.1074/jbc.M113.528968 . 24692550 10.1074/jbc.M113.528968 PMC4036341

[20] Magalhaes PJ, Andreu AL, Schon EA. Evidence for the presence of 5S rRNA in mammalian mitochondria. Molecular biology of the cell. 1998;9(9):2375–82. . 9725900 10.1091/mbc.9.9.2375 PMC25503

[21] Entelis NS, Kolesnikova OA, Dogan S, Martin RP, Tarassov IA. 5 S rRNA and tRNA import into human mitochondria. Comparison of in vitro requirements. J Biol Chem. 2001;276(49):45642–53. doi: 10.1074/jbc.M103906200 . 11551911 10.1074/jbc.M103906200

[22] Yoshionari S, Koike T, Yokogawa T, Nishikawa K, Ueda T, Miura K, et al Existence of nuclear-encoded 5S-rRNA in bovine mitochondria. FEBS letters. 1994;338(2):137–42. . 7508404 10.1016/0014-5793(94)80351-x

[23] Greber BJ, Ban N. Structure and Function of the Mitochondrial Ribosome. Annual review of biochemistry. 2016;85:103–32. doi: 10.1146/annurev-biochem-060815-014343 . 27023846 10.1146/annurev-biochem-060815-014343

[24] Smirnov A, Comte C, Mager-Heckel AM, Addis V, Krasheninnikov IA, Martin RP, et al Mitochondrial enzyme rhodanese is essential for 5 S ribosomal RNA import into human mitochondria. J Biol Chem. 2010;285(40):30792–803. doi: 10.1074/jbc.M110.151183 . 20663881 10.1074/jbc.M110.151183 PMC2945573

[25] Smirnov A, Entelis N, Martin RP, Tarassov I. Biological significance of 5S rRNA import into human mitochondria: role of ribosomal protein MRP-L18. Genes & development. 2011;25(12):1289–305. doi: 10.1101/gad.624711 . 21685364 10.1101/gad.624711 PMC3127430

[26] Smirnov A, Tarassov I, Mager-Heckel AM, Letzelter M, Martin RP, Krasheninnikov IA, et al Two distinct structural elements of 5S rRNA are needed for its import into human mitochondria. Rna. 2008;14(4):749–59. doi: 10.1261/rna.952208 . 18314502 10.1261/rna.952208 PMC2271358

[27] Dovydenko I, Heckel AM, Tonin Y, Gowher A, Venyaminova A, Tarassov I, et al Mitochondrial targeting of recombinant RNA. Methods Mol Biol. 2015;1265:209–25. doi: 10.1007/978-1-4939-2288-8_16 . 25634278 10.1007/978-1-4939-2288-8_16

[28] Mercer TR, Neph S, Dinger ME, Crawford J, Smith MA, Shearwood AM, et al The human mitochondrial transcriptome. Cell. 2011;146(4):645–58. doi: 10.1016/j.cell.2011.06.051 . 21854988 10.1016/j.cell.2011.06.051 PMC3160626

[29] Schneider A. Mitochondrial tRNA import and its consequences for mitochondrial translation. Annual review of biochemistry. 2011;80:1033–53. doi: 10.1146/annurev-biochem-060109-092838 . 21417719 10.1146/annurev-biochem-060109-092838

[30] Towheed A, Markantone DM, Crain AT, Celotto AM, Palladino MJ. Small mitochondrial-targeted RNAs modulate endogenous mitochondrial protein expression in vivo. Neurobiology of disease. 2014;69:15–22. doi: 10.1016/j.nbd.2014.04.017 . 24807207 10.1016/j.nbd.2014.04.017 PMC4106415

[31] Amunts A, Brown A, Bai XC, Llacer JL, Hussain T, Emsley P, et al Structure of the yeast mitochondrial large ribosomal subunit. Science. 2014;343(6178):1485–9. doi: 10.1126/science.1249410 . 24675956 10.1126/science.1249410 PMC4046073

[32] Greber BJ, Boehringer D, Leibundgut M, Bieri P, Leitner A, Schmitz N, et al The complete structure of the large subunit of the mammalian mitochondrial ribosome. Nature. 2014;515(7526):283–6. doi: 10.1038/nature13895 . 25271403 10.1038/nature13895

[33] Gammage PA, Moraes CT, Minczuk M. Mitochondrial Genome Engineering: The Revolution May Not Be CRISPR-Ized. Trends in genetics: TIG. 2017 doi: 10.1016/j.tig.2017.11.001 . 29179920 10.1016/j.tig.2017.11.001 PMC5783712

[34] Chrzanowska-Lightowlers Z, Rorbach J, Minczuk M. Human mitochondrial ribosomes can switch structural tRNAs—but when and why? RNA Biol. 2017;14(12):1668–71. doi: 10.1080/15476286.2017.1356551 . 28786741 10.1080/15476286.2017.1356551 PMC5731804

[35] Karicheva OZ, Kolesnikova OA, Schirtz T, Vysokikh MY, Mager-Heckel AM, Lombes A, et al Correction of the consequences of mitochondrial 3243A>G mutation in the MT-TL1 gene causing the MELAS syndrome by tRNA import into mitochondria. Nucleic Acids Res. 2011;39(18):8173–86. doi: 10.1093/nar/gkr546 . 21724600 10.1093/nar/gkr546 PMC3185436

[36] Dovydenko I, Tarassov I, Venyaminova A, Entelis N. Method of carrier-free delivery of therapeutic RNA importable into human mitochondria: Lipophilic conjugates with cleavable bonds. Biomaterials. 2016;76:408–17. doi: 10.1016/j.biomaterials.2015.10.075 . 26561937 10.1016/j.biomaterials.2015.10.075

[37] Zelenka J, Alan L, Jaburek M, Jezek P. Import of desired nucleic acid sequences using addressing motif of mitochondrial ribosomal 5S-rRNA for fluorescent in vivo hybridization of mitochondrial DNA and RNA. Journal of bioenergetics and biomembranes. 2014;46(2):147–56. doi: 10.1007/s10863-014-9543-2 . 24562889 10.1007/s10863-014-9543-2

[38] Autour A, S CYJ, A DC, Abdolahzadeh A, Galli A, Panchapakesan SSS, et al Fluorogenic RNA Mango aptamers for imaging small non-coding RNAs in mammalian cells. Nature communications. 2018;9(1):656 doi: 10.1038/s41467-018-02993-8 . 29440634 10.1038/s41467-018-02993-8 PMC5811451

[39] Pohjoismaki JL, Goffart S. Of circles, forks and humanity: Topological organisation and replication of mammalian mitochondrial DNA. BioEssays: news and reviews in molecular, cellular and developmental biology. 2011;33(4):290–9. doi: 10.1002/bies.201000137 . 21290399 10.1002/bies.201000137

[40] Clayton DA. Mitochondrial DNA replication: what we know. IUBMB life. 2003;55(4–5):213–7. doi: 10.1080/1521654031000134824 . 12880201 10.1080/1521654031000134824

[41] Yasukawa T, Reyes A, Cluett TJ, Yang MY, Bowmaker M, Jacobs HT, et al Replication of vertebrate mitochondrial DNA entails transient ribonucleotide incorporation throughout the lagging strand. The EMBO journal. 2006;25(22):5358–71. doi: 10.1038/sj.emboj.7601392 . 17066082 10.1038/sj.emboj.7601392 PMC1636616

[42] Bowmaker M, Yang MY, Yasukawa T, Reyes A, Jacobs HT, Huberman JA, et al Mammalian mitochondrial DNA replicates bidirectionally from an initiation zone. J Biol Chem. 2003;278(51):50961–9. doi: 10.1074/jbc.M308028200 . 14506235 10.1074/jbc.M308028200

[43] Herbers E, Kekalainen NJ, Hangas A, Pohjoismaki JL, Goffart S. Tissue specific differences in mitochondrial DNA maintenance and expression. Mitochondrion. 2018 doi: 10.1016/j.mito.2018.01.004 . 29339192 10.1016/j.mito.2018.01.004

[44] Phillips AF, Millet AR, Tigano M, Dubois SM, Crimmins H, Babin L, et al Single-Molecule Analysis of mtDNA Replication Uncovers the Basis of the Common Deletion. Molecular cell. 2017;65(3):527–38.e6. doi: 10.1016/j.molcel.2016.12.014 . 28111015 10.1016/j.molcel.2016.12.014

[45] Chretien D, Benit P, Ha HH, Keipert S, El-Khoury R, Chang YT, et al Mitochondria are physiologically maintained at close to 50 degrees C. PLoS biology. 2018;16(1):e2003992 doi: 10.1371/journal.pbio.2003992 . 29370167 10.1371/journal.pbio.2003992 PMC5784887

[46] Marroquin LD, Hynes J, Dykens JA, Jamieson JD, Will Y. Circumventing the Crabtree effect: replacing media glucose with galactose increases susceptibility of HepG2 cells to mitochondrial toxicants. Toxicological sciences: an official journal of the Society of Toxicology. 2007;97(2):539–47. doi: 10.1093/toxsci/kfm052 . 17361016 10.1093/toxsci/kfm052

[47] King MP, Attardi G. Human cells lacking mtDNA: repopulation with exogenous mitochondria by complementation. Science. 1989;246(4929):500–3. . 2814477 10.1126/science.2814477

[48] Dott W, Mistry P, Wright J, Cain K, Herbert KE. Modulation of mitochondrial bioenergetics in a skeletal muscle cell line model of mitochondrial toxicity. Redox biology. 2014;2:224–33. doi: 10.1016/j.redox.2013.12.028 . 24494197 10.1016/j.redox.2013.12.028 PMC3909783

[49] Elkalaf M, Andel M, Trnka J. Low glucose but not galactose enhances oxidative mitochondrial metabolism in C2C12 myoblasts and myotubes. PloS one. 2013;8(8):e70772 doi: 10.1371/journal.pone.0070772 . 23940640 10.1371/journal.pone.0070772 PMC3733643

[50] Cannino G, El-Khoury R, Pirinen M, Hutz B, Rustin P, Jacobs HT, et al Glucose modulates respiratory complex I activity in response to acute mitochondrial dysfunction. J Biol Chem. 2012;287(46):38729–40. doi: 10.1074/jbc.M112.386060 . 23007390 10.1074/jbc.M112.386060 PMC3493916

[51] Santra S, Gilkerson RW, Davidson M, Schon EA. Ketogenic treatment reduces deleted mitochondrial DNAs in cultured human cells. Ann Neurol. 2004;56(5):662–9. doi: 10.1002/ana.20240 . 15389892 10.1002/ana.20240

[52] Desquiret-Dumas V, Gueguen N, Barth M, Chevrollier A, Hancock S, Wallace DC, et al Metabolically induced heteroplasmy shifting and l-arginine treatment reduce the energetic defect in a neuronal-like model of MELAS. Biochimica et biophysica acta. 2012;1822(6):1019–29. doi: 10.1016/j.bbadis.2012.01.010 . 22306605 10.1016/j.bbadis.2012.01.010 PMC3339237

[53] Frey S, Geffroy G, Desquiret-Dumas V, Gueguen N, Bris C, Belal S, et al The addition of ketone bodies alleviates mitochondrial dysfunction by restoring complex I assembly in a MELAS cellular model. Biochimica et biophysica acta. 2017;1863(1):284–91. doi: 10.1016/j.bbadis.2016.10.028 . 27815040 10.1016/j.bbadis.2016.10.028

[54] Markham NR, Zuker M. UNAFold: software for nucleic acid folding and hybridization. Methods Mol Biol. 2008;453:3–31. doi: 10.1007/978-1-60327-429-6_1 . 18712296 10.1007/978-1-60327-429-6_1

[55] Szymanski M, Barciszewska MZ, Erdmann VA, Barciszewski J. 5 S rRNA: structure and interactions. The Biochemical journal. 2003;371(Pt 3):641–51. doi: 10.1042/BJ20020872 . 12564956 10.1042/BJ20020872 PMC1223345

[56] Sugimoto N, Nakano S, Katoh M, Matsumura A, Nakamuta H, Ohmichi T, et al Thermodynamic parameters to predict stability of RNA/DNA hybrid duplexes. Biochemistry. 1995;34(35):11211–6. . 7545436 10.1021/bi00035a029

