# Supplementary figures and images for: Anti-replicative recombinant 5S rRNA molecules can modulate the mtDNA heteroplasmy in a glucose-dependent manner
Source: PLoS One. 2018 Jun 18;13(6):e0199258. doi: 10.1371/journal.pone.0199258 (PMC6005506; doi:10.1371/journal.pone.0199258)

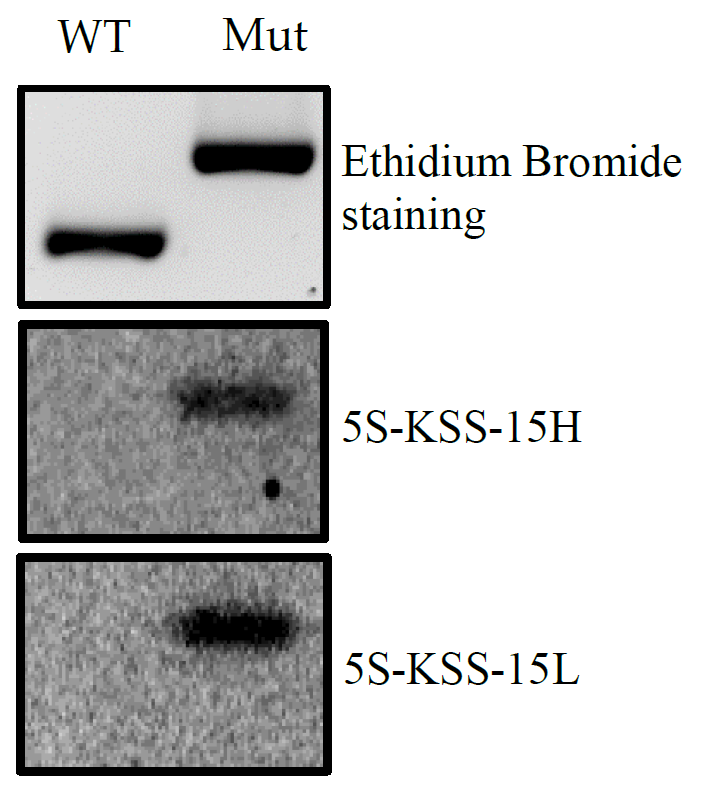

Supplement: S1 Fig — Southern hybridization of wild-type (WT) or KSS mtDNA fragments (Mut) with 32P-labelled rec.5S rRNAs “5S-KSS-15H” and “5S-KSS-15L” (as indicated at the right) at 37°C in 1xPBS. (TIF) [file pone.0199258.s001.tif]

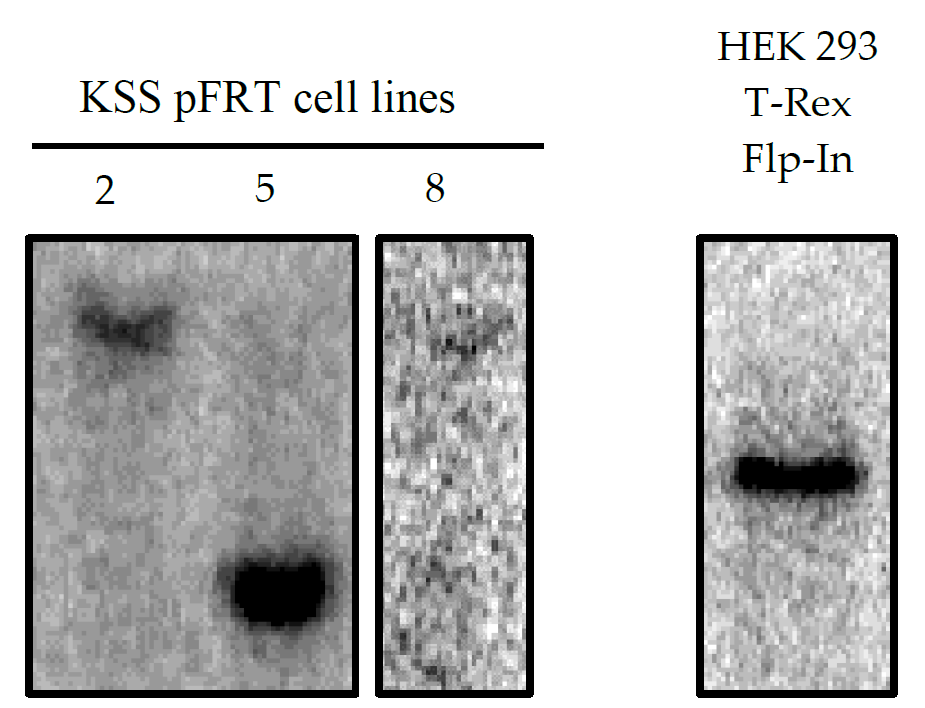

Supplement: S3 Fig — Southern blot hybridization of genomic DNA from three KSS-FRT clones (2, 5 and 8) compared to commercial HEK 293 T-Rex™ Flp-In™ cells. (TIF) [file pone.0199258.s003.tif]

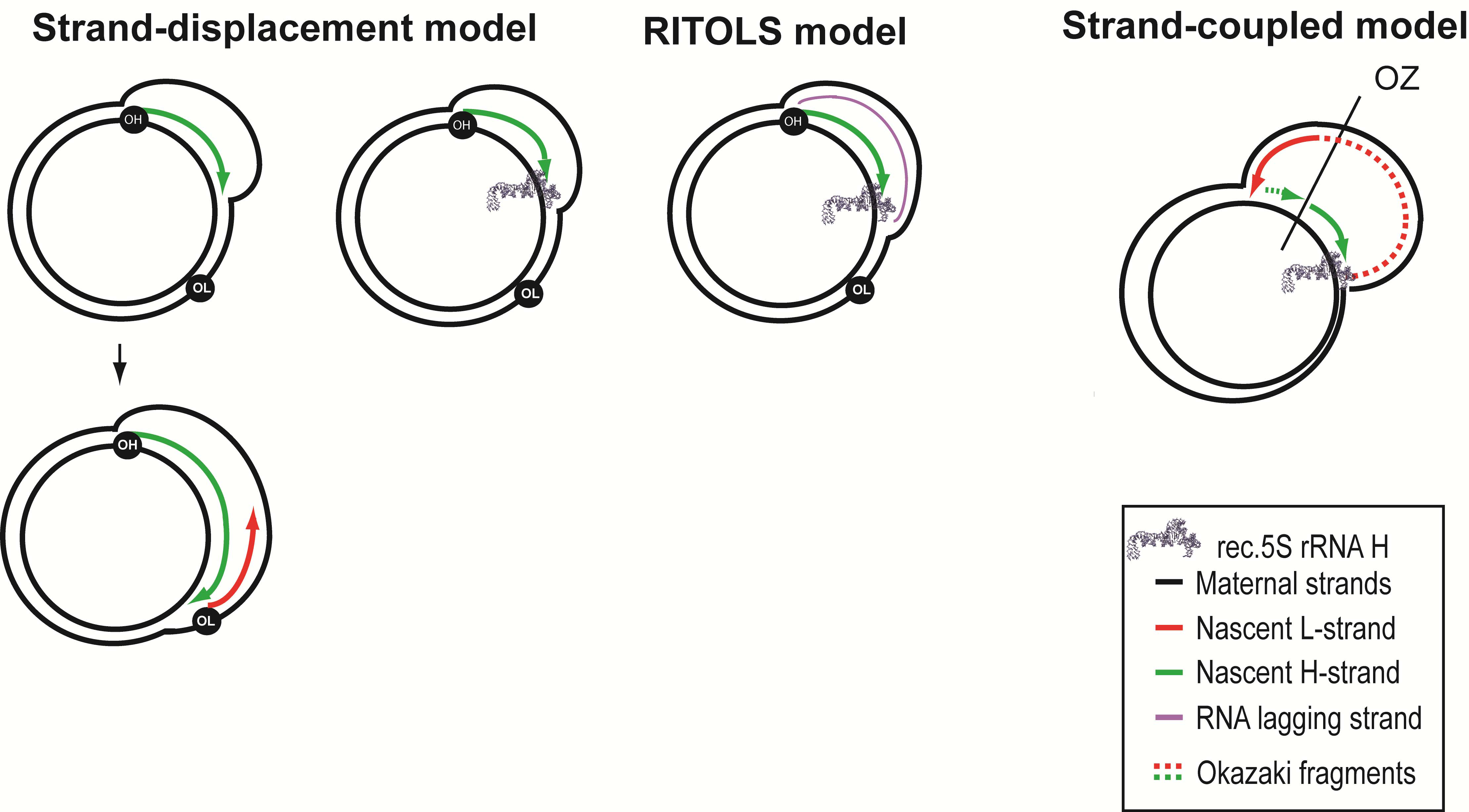

Supplement: S4 Fig — (TIF) [file pone.0199258.s004.tif]
