# Supplementary material for: Anti-replicative recombinant 5S rRNA molecules can modulate the mtDNA heteroplasmy in a glucose-dependent manner
Source: PLoS One. 2018 Jun 18;13(6):e0199258. doi: 10.1371/journal.pone.0199258 (PMC6005506; doi:10.1371/journal.pone.0199258)
Supplement: S2 Fig — Northern blot analysis of rec.5S rRNA variants in total and mitoplast RNA preparations from cells transfected with various RNA (as indicated above the panels). Originals gel stained with Ethidium bromide (EthBr) and hybridized with probes indicated at the left, as on Fig 3. (DOCX) [file pone.0199258.s002.docx]

**S2 Figure. *In vivo* test of rec.5S rRNA import into human mitochondria.** Northern blot analysis of rec.5S rRNA variants in total and mitoplast RNA preparations from cells transfected with various RNA (as indicated above the panels). Originals gel stained with Ethidium bromide (EthBr) and hybridized with probes indicated at the left, as on Fig 3.


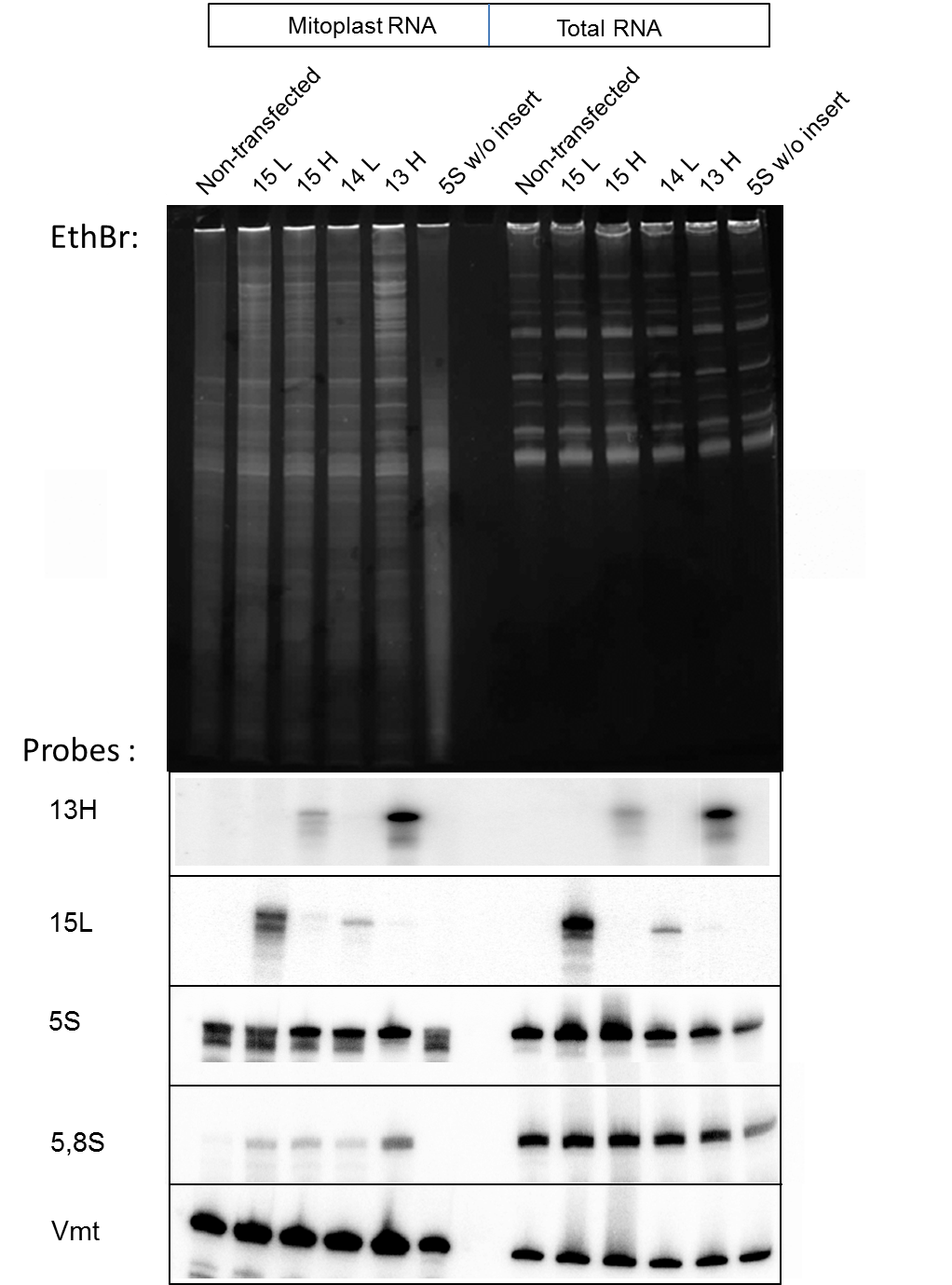


Probe 13H can hybridize with rec.5S rRNA-KSS-13H and rec.5S rRNA-KSS-15H, the signal for rec.5S rRNA-KSS-15H is weaker due to a mismatch; the same is for the probe 15L (hybridization with rec.5S rRNA-KSS-14L is weaker than with rec.5S rRNA-KSS-15L). 5S w/o insert corresponds to cells transfected with wild type 5S rRNA transcript; in this case RNA import was not quantified due to important degradation of mitoplast RNA. Probing with 5.8S rRNA revealed that the mitoplast RNA from cells transfected with rec.5S rRNA-KSS-13H was contaminated with cytosolic RNAs, this sample could not be quantified; another transfection experiment is shown on Fig 3.
