# Supplementary material for: Anti-replicative recombinant 5S rRNA molecules can modulate the mtDNA heteroplasmy in a glucose-dependent manner
Source: PLoS One. 2018 Jun 18;13(6):e0199258. doi: 10.1371/journal.pone.0199258 (PMC6005506; doi:10.1371/journal.pone.0199258)
Supplement: S1 Table — (DOCX) [file pone.0199258.s005.docx]

**S1 Table.** List of oligonucleotide primers used in the study

| N° | Name | Sequence |
| --- | --- | --- |
| 1 | T7-prom | 5' GGGATCCATAATACGACTCACTATA 3' |
| 2 | KSS-13H-rev | 5’ AGGCCCGACCCTGCTTAGCTACAGTGCTTACTTTTCAGGGTGGTATGGCCGTA 3’ |
| 3 | KSS-14L-rev | 5’ AGGCCCGACCCTGCTTAGCTAAGTAAGCACTGTATTCAGGGTGGTATGGCCGTA 3’ |
| 4 | KSS-15H-rev | 5’ AGGCCCGACCCTGCTTAGCTTTACAGTGCTTACTTTTCAGGGTGGTATGGCCGTA 3’ |
| 5 | KSS-15L-rev | 5’ AGGCCCGACCCTGCTTAGCTAGAAGTAAGCACTGTTTCAGGGTGGTATGGCCGTA 3’ |
| 6 | 5S-BglII | 5’ GGAGATCTAAGCCTACAACACCCGG 3’ |
| 7 | Cytbwt F | 5' CTTTAAAGCTTCACACGATTCTTTACCTTTC 3' |
| 8 | Cytbwt R | 5' TCTTTGGAATTCGTTTGGATATATGGAGGATGG 3' |
| 9 | Lysmt F | 5' TCATACAAGCTTACAGATGCAATTCCCGGACG 3' |
| 10 | 5'-HindIII-5S | 5' CCAAGCTTGGGCGGGGCTGGGCTCTTGGGGCAGCCAGGCGCCTCCTTCAGCGCCTACGGCCATACCACCC 3' |
| 11 | 3'-BamHI-5S | 5' CCGGATCCAAAGCCAAAGAAAAGCCTACAACACCCG 3' |
| 12 | 5'-BamHI-5S | 5'CCGGATCCGGGCGGGGCTGGGCTCTTGGGGCAGCCAGGCGCCTCCTTCAGCGCCTACGGCCATACCACCC 3' |
| 13 | 3'-HindIII-5S | 5' CCAAGCTTAAAGCCAAAGAAAAGCCTACAACACCCG 3' |
| 14 | 1095 F | 5' TAGCCCTAAACCTCAACAGT 3' |
| 15 | 1305 B | 5' TGCGCTTACTTTGTAGCCTTCAT 3' |
| 16 | 11614 F | 5' CATTGCATACTCTTCAATCAGC 3' |
| 17 | 11778 B | 5' CGACTGTGAGTGCGTT 3' |
| 18 | RT-5S-KSS-H | 5' CACCCTGAAAAGTAAGCAC 3' |
| 19 | RT-5S-KSS-L | 5' CCTGAATACAGTGCTTAC 3' |
| 20 | RT-15H-KSS | 5' GAGAAGTAAGCACTGTAAAG 3' |
| 21 | RT-15L-KSS | 5' CCCTGAAACAGTGCTTAC 3' |
| 22 | RT-5S-Antisens | 5' AAGCCTACAACACCC 3' |
| 23 | TST1-For | 5' GTGGATGTTCCGTGTGTTTGG 3' |
| 24 | TST1-Rev | 5' CAGCACCTGCTCGTAGGTC 3' |
| 25 | Anti-insert 13H,15H | 5' GCTTACAGTGCTTACTT 3' |
| 26 | Anti-insert 14L,15L | 5' AAGTAAGCACTGTTTCAG 3' |
| 27 | Mt tRNA Valine | 5' TGGGTCAGAGCGGTCAAGTTAAGTTGAAATCTCC 3' |
| 28 | Anti-5,8S rRNA | 5' GGCCGCAAGTGCGTTCGAAG 3' |
| 29 | Anti-5S rRNA | 5' AAAGCCTACAACACCCGGTATTCCC 3' |
