# Supplementary material for: Anti-replicative recombinant 5S rRNA molecules can modulate the mtDNA heteroplasmy in a glucose-dependent manner
Source: PLoS One. 2018 Jun 18;13(6):e0199258. doi: 10.1371/journal.pone.0199258 (PMC6005506; doi:10.1371/journal.pone.0199258)
Supplement: S2 Table — (DOCX) [file pone.0199258.s006.docx]

Endogenous 5S rRNA import was analyzed in non-transfected cells and taken as 100%. Data on the endogenous 5S import efficiency for the individual transient transfections are not shown due to cross-hybridization of the 5S probe with rec.5S rRNA molecules.
