# Supplementary material for: Anti-replicative recombinant 5S rRNA molecules can modulate the mtDNA heteroplasmy in a glucose-dependent manner
Source: PLoS One. 2018 Jun 18;13(6):e0199258. doi: 10.1371/journal.pone.0199258 (PMC6005506; doi:10.1371/journal.pone.0199258)
Supplement: S3 Table — (DOCX) [file pone.0199258.s007.docx]

**S3 Table**. Characteristics of the three independent *trans*mitochondrial KSS-FRT cell lines (numbered 2, 5 and 8) compared to the commercial cells HEK 293 T-Rex^TM^ Flp-In^TM^.

| KSS pFRT cell line | KSS pFRT 2 | KSS pFRT 5 | KSS pFRT 8 | HEK 293 T-Rex^TM^ Flp-In^TM^ |
| --- | --- | --- | --- | --- |
| Number of FRT site | 1 | 1 | 1 | 1 |
| β-gal Specific Activity (in nmol of hydrolyzed ONPG/30 min/protein mg) | 23 ±10 | 31 ±3 | 15 ±6 | 125 ±6 |
| KSS heteroplasmy level | 52 ±5% | 51 ±4% | 52 ±5% | / |
