# Supplementary material for: Anti-replicative recombinant 5S rRNA molecules can modulate the mtDNA heteroplasmy in a glucose-dependent manner
Source: PLoS One. 2018 Jun 18;13(6):e0199258. doi: 10.1371/journal.pone.0199258 (PMC6005506; doi:10.1371/journal.pone.0199258)
Supplement: S4 Table — (DOCX) [file pone.0199258.s008.docx]

**S4 Table.** Quantification of anti-replicative molecules in rec.5S rRNA expressing cell lines by two approaches: semi-quantitative one step RT-PCR and two steps real-time RT-qPCR.

| **Rec.5S rRNA** | Number rec.5S rRNA/cell  RT-PCR semi-quant. | Number rec.5S rRNA/cell  real time RT-qPCR |
| --- | --- | --- |
| 5S-KSS-13H**^(1)^** | 2000±200 | 740±8 |
| 5S-KSS-13H | 200±30 | 75±3 |
| 5S-KSS-14L^(^**^1^**^)^ | 2000±200 | ND |
| 5S-KSS-14L | 300±50 | 120±5 |
| 5S-KSS-15H | 1300±200 | 500±5 |
| 5S-KSS-15L | 200±30 | 70±3 |

Values obtained by two steps RT-qPCR are ≈ three fold decreased compared to those of semi-quantitative RT-PCR. This can be explained by two factors: 1) different efficiency of cDNA synthesis, since different enzymes had been used for the One-step and for two steps RT-PCR reactions; 2) discrepancy of cell quantification mode in experiments performed now and a year ago. Despite the difference in absolute values, we obtained the same proportion of the rec.5S rRNA expression among the cell lines: the best expression for 5S-KSS-13H^(1)^, lower for 5S-KSS-15H and rather small expression for other lines. Thus, we believe that new RT-qPCR data do not change the main claims of our study.
